# Naturalistic comparison of clomethiazole and Diazepam treatment in alcohol withdrawal: effects on oxidative stress, inflammatory cytokines and hepatic biomarkers

**DOI:** 10.1007/s00406-024-01835-7

**Published:** 2024-06-08

**Authors:** David Strischewski, Amira Kalmar, Paul C. Guest, Henrik Dobrowolny, Gabriela Meyer-Lotz, Conrad J. Schiffner, Wolfgang Jordan, Ulf J. Müller, Katrin Borucki, Michael Böttcher, Borna Relja, Johann Steiner

**Affiliations:** 1 Department of Psychiatry, Otto-Von-Guericke-University Magdeburg, Leipziger Str. 44, 39120 Magdeburg, Germany; 2 Laboratory of Translational Psychiatry, Otto-Von-Guericke-University Magdeburg, Magdeburg, Germany; 3 Department of Anaesthesiology and Intensive Care Medicine, Klinikum Chemnitz, Chemnitz, Germany; 4 Laboratory of Neuroproteomics, Department of Biochemistry and Tissue Biology, Institute of Biology, University of Campinas (UNICAMP), Campinas, Brazil; 5 Department of Anesthesiology and Intensive Care Medicine, Otto-Von-Guericke-University Magdeburg, Magdeburg, Germany; 6 Department of Psychiatry and Psychotherapy, Magdeburg Hospital GmbH, Magdeburg, Germany; 7 Department of Psychiatry and Psychotherapy, University of Goettingen, Goettingen, Germany; 8 Forensic Psychiatric State Hospital of Saxony-Anhalt, Stendal-Uchtspringe, Germany; 9 Institute of Clinical Chemistry and Pathobiochemistry, Otto-Von-Guericke-University Magdeburg, Magdeburg, Germany; 10 MVZ Medizinische Labore Dessau Kassel GmbH, Dessau-Rosslau, Germany; 11 Department of Trauma, Hand, Plastic and Reconstructive Surgery, Translational and Experimental Trauma Research, University Hospital Ulm, University Ulm, Ulm, Germany; 12 Center for Behavioral Brain Sciences (CBBS), Magdeburg, Germany; 13 Center for Health and Medical Prevention (CHaMP), Magdeburg, Germany; 14 German Center for Mental Health (DZPG), Partner Site Halle-Jena-Magdeburg, Magdeburg, Germany

**Keywords:** Alcohol withdrawal treatment, Alcohol detoxification, Clomethiazole, Diazepam, Dityrosine, Malondialdehyde, Carbohydrate-deficient transferrin, Ethyl glucuronide, Phosphatidylethanol, Inflammation, Transaminases

## Abstract

Ethanol is metabolized by alcohol dehydrogenase to acetaldehyde and induces cytochrome P450 2E1 (CYP2E1), which generates reactive oxygen species that cause inflammatory liver damage. Clomethiazole, a drug approved for alcohol withdrawal treatment (AWT) in some European countries, inhibits CYP2E1. We hypothesized that clomethiazole would lead to a faster reduction in oxidative stress, inflammatory cytokines, and liver enzymes compared to diazepam treatment. We analysed respective biomarkers in 50 patients undergoing AWT and 25 healthy individuals but found no statistical difference between the two medication groups over 3–5 days. Hence, our hypothesis was not confirmed during this observation period.

**Supplementary Information:**

The online version contains supplementary material available at 10.1007/s00406-024-01835-7.

## Introduction

Alcohol addiction remains a significant global public health concern, with a global prevalence rate of around 3 per cent, which varies by region [1]. Worldwide, 3 million deaths per year result from harmful use of alcohol, representing 5.3% of all deaths. Beyond health consequences, the harmful use of alcohol brings significant social and economic losses to individuals and society. Nevertheless, the availability and coverage of alcohol withdrawal treatment is limited in many parts of the world [1].

Chronic alcohol consumption of more than 40 g per day induces cytochrome P450 2E1 (CYP2E1), which enhances its own oxidation to acetaldehyde [2]. Structural cellular and mitochondrial alterations by acetaldehyde cause decreased adenosine triphosphate generation and production of reactive oxygen species (ROS). These cause damage to macromolecules, including lipid peroxidation and protein oxidation, with generation of oxidation products such as malondialdehyde (MDA) and dityrosine, and production of proinflammatory cytokines that activate neutrophils and induce liver damage [2]. In Germany, alcohol withdrawal treatment (AWT) is administered mainly as inpatient therapy. National guidelines generally recommend use of symptom-based protocols, which tend to have shorter treatment durations [3, 4]. Clomethiazole (CMZ) is the only drug approved for AWT in Germany.^1^ Nevertheless, many healthcare providers use benzodiazepines such as diazepam (DIAZ), although a network meta-analysis by Bahji et al. has shown that CMZ appears to be superior to DZP and some other benzodiazepines in preventing seizures and reducing the severity of alcohol withdrawal [5]. The finding that CMZ inhibits CYP2E1 [6], suggests that AWT with CMZ might result in less oxidative stress and inflammation, and hence quicker normalization of liver parameters compared to treatment with benzodiazepines [7, 8].

Our naturalistic prospective study aimed to assess the benefits of either CMZ or DIAZ in AWT, by applying longitudinal measurements of biomarkers for oxidative stress, inflammation and liver injury in patients.

## Methods

### Study population

From October 2017 to August 2020, patients (18–65 years-old) routinely admitted for AWT were screened for participation in this prospective study. Ethics approval was obtained from the Otto-von-Guericke-University Magdeburg Institutional Review Board (reference number 80/17). We applied ICD-10 criteria to diagnose alcohol dependence and the Alcohol Use Disorders Identification Test (AUDIT) was used as a supporting test [9]. Alcohol craving was measured with the Obsessive Compulsive Drinking Scale (OCDS) [10]. Exclusion criteria encompassed concomitant immunological diseases, severe infections, trauma, terminal illnesses affecting the brain (e.g. severe liver, kidney, heart and lung diseases, and tumours), treatment with cortisone or other immunosuppressive or immunomodulating substances, co-use of psychotropic substances, and incapacity or refusal for additional blood tests. In this study, the choice of drug was naturalistic and the study was prospective, with CMZ and DIAZ being administered using symptom-based protocols. A study sketch was created which was approved by the ethics committee and the patients who came to our department for AWT gave written informed consent to be willing to allow longitudinal blood takes, willingness to answer clinical questionnaires and for analysis of data from rating scales. Two treatment groups of 25 patients were established, matched for sex, body mass index (BMI), age, smoking, chronic obstructive pulmonary disease (COPD), and age at first AWT (Table 1; [4, 11]).^2^ Control samples (n = 25) were obtained during the same period from healthy donors, including hospital staff, students and their relatives.

**Table 1:** Demographic, clinical and routine laboratory data at baseline

Variables	CMZ (n = 25)	DIAZ (n = 25)	Controls (n = 25)	Test	Test value	p-value	Post-hoc tests (p-value, FDR)		
							DIAZ-CMZ	CMZ-Controls	DIAZ-Controls
Age (y)	51.0 (42.0;57.0)	51.0 (38.0;56.5)	53.0 (43.0;56.5)	H-test	KW = 0.26	0.878	0.838	0.838	0.838
Sex (female/male)	5/20	4/21	4/21	Fisher exact	–	1.000	1.000	1.000	1.000
BMI (kg/m^2^)	24.6 (22.0;27.0)	24.0 (19.6;26.7)	25.7 (24.0;28.3)	H-test	KW = 4.43	0.109	0.453	0.204	0.168
Smoking (yes/no)	19/6	22/3	2/23	Fisher exact	–	**<0.001**	0.463	**<0.001**	**<0.001**
COPD (yes/no)	Yes: 3/No: 22	Yes: 4/No: 21	Yes: 0/No: 0	Fisher exact	–	1.000	1.000	1.000	1.000
Age at first alcohol withdrawal treatment (y)	41.0 (29.3;44.5)	32.0 (24.5;49.5)	–	U-Test	W = 102.00	0.948			
AUDIT (sum score)	27.0 (22.0;31.0)	29.0 (23.5;33rr.0)	2.0 (0.0;3.0)	H-test	KW = 46.17	**<0.001**	0.517	**<0.001**	**<0.001**
OCDS (sum score)	19.0 (15.0;23.0)	21.0 (14.8;27.0)	1.0 (0.0;1.0)	H-test	KW = 47.40	**<0.001**	0.562	**<0.001**	**<0.001**
Leukocyte count (× 10^9^/L)	7.60 (6.12;8.74)	7.00 (6.00;9.00)	6.77 (5.94;7.40)	H-test	KW = 3.71	0.157	0.749	0.134	0.355
CRP (mg/L)	0.70 (0.25;4.01)	2.32 (0.60;5.85)	0.95 (0.63;2.45)	H-test	KW = 3.93	0.140	0.181	0.467	0.181
Biomarkers of alcohol consumption and liver damage									
BAC (neg/pos)	7/18	6/19	25/0	Fisher exact	-	**<0.001**	1.000	**<0.001**	**<0.001**
BAC (^o^/_oo_, only for BAC pos cases)	1.9 (0.9;2.6)	2.1 (1.4;2.5)	–	U-test	W = 144.50	0.429			
EtG in whole blood (neg/pos)	0/25	1/24	19/6	Fisher exact	-	**<0.001**	1.000	**<0.001**	**<0.001**
EtG in whole blood (ng/mL)	1736 (590;4158)	3355 (1371;4820)	5 (2;23)	H-test	KW = 14.67	**<0.001**	0.298	**<0.001**	**<0.001**
PEth in whole blood (neg/pos)	0/25	0/25	12/11	Fisher exact	-	**<0.001**	1.000	**<0.001**	**<0.001**
PEth in whole blood (µmol/L)	4.69 (3.44;6.14)	4.28 (3.12;6.24)	0.09 (0.07;0.14)	H-test	KW = 26.53	**<0.001**	0.882	**<0.001**	**<0.001**
CDT (neg/pos)	6/18	6/19	23/0	Fisher exact	-	**<0.001**	1.000	**<0.001**	**<0.001**
CDT (%)	5.1 (3.0;9.9)	6.4 (3.7;10.1)	–	U-test	W = 167.00	0.915			
GGT (µmol/L)	2.44 (1.25;7.91)	1.96 (0.73;3.01)	0.38 (0.29;0.56)	H-test	KW = 31.01	**<0.001**	0.187	**<0.001**	**<0.001**
ALAT (µmol/L)	0.84 (0.55;1.31)	0.84 (0.53;0.96)	0.36 (0.30;0.46)	H-test	KW = 25.30	**<0.001**	0.438	**<0.001**	**<0.001**
ASAT (µmol/L)	1.08 (0.77;1.64)	1.10 (0.63;2.16)	0.41 (0.37;0.47)	H-test	KW = 41.89	**<0.001**	0.915	**<0.001**	**<0.001**
ASAT/ALAT ratio	1.29 (1.05;1.92)	1.43 (1.15;2.01)	1.08 (0.90;1.28)	H-test	KW = 11.31	**0.004**	0.528	**0.027**	**0.003**
MCV (fl)	93.7 (90.1;97.5)	91.0 (87.8;95.9)	87.8 (83.8;91.4)	H-test	KW = 18.47	**<0.001**	0.114	**<0.001**	**0.013**

Data are presented as median (quartile 1; quartile 3) or number of cases

*p*-values in bold are statistically significant

*FDR* false discovery rate, *neg* negative, *pos* positive

### Samples and analyses

EDTA-blood, serum and urine samples were obtained upon admission before medication initiation (baseline, BL) and on days 3 (D3) and 5 (D5). All participants underwent a urine drug screen. A complete differential blood count was determined using an XN-3000 automated counter (Sysmex Corporation; Kobe, Japan). Measurements of C-reactive protein (CRP), creatinine, glucose, lipids, and *biomarkers of liver damage* [alanine-aminotransferase (ALAT), aspartate-aminotransferase (ASAT), gamma-glutamyltransferase (GGT)] were determined on a random access nalyser (cobas c701/c501, Roche Diagnostics; Mannheim, Germany).

*Markers of alcohol consumption* at admission included blood alcohol concentration (BAC: cut-off 0.1°/_oo_, enzymatic alcohol dehydrogenase/ADH oxidation), ethyl glucuronide (EtG: cut-off 1 ng/mL) [12], phosphatidylethanol (Peth: cut-off 0.050 µmol/L) [13], and carbohydrate-deficient transferrin (CDT: cut-off 2.0%, high performance liquid chromatography/HPLC, ClinRep®, RECIPE Chemicals + Instruments; GmbH, Germany).

*Blood CMZ concentrations* were determined on an Acquity Ultra Performance Liquid Chromatograph connected to a Xevo-XS tandem mass spectrometer using the Chromsystems [Waters; Munich, Germany; limit of quantification (LOQ) 15 ng/mL]. *Diazepam* and the active metabolite *Nordiazepam* were quantified using the same system (LOQ 1 ng/mL).

As indicator of *oxidative stress*, total *MDA* in plasma samples was quantified through derivatization with 2–4-dinitrophenylhydrazine, and urinary *dityrosine* levels was measured using HPLC with fluorimetric detection following solid-phase extraction (Macherey & Nagel, Germany), as described [14]. Serum concentrations of 17 *inflammatory cytokines* (Table 2) were assessed using the LEGENDplex™ Human Inflammation Panel 1 multiplex immunoassay (BioLegend, San Diego, USA).

**Table 2:** Linear Mixed Model (LMM) analyses of liver enzymes, MDA, dityrosine or cytokines as the respective dependent variables, drug type (CMZ or DIAZ) and time point (baseline, day 3, day 5) as fixed factors, and blood alcohol concentration (BAC) on admission as a covariate

Variables	Influence of drug type (p-value)	Influence of time (p-value)	Influence of BAC on admission (p-value)	Interaction drug type X time (p-value)
GGT (µmol/L)	0.616	0.050	0.647	0.368
ALAT (µmol/L)	0.841	0.113	0.482	0.069
ASAT (µmol/L)	0.709	0.567	0.092	0.129
ASAT/ALAT ratio	0.792	**<0.001**	**0.040**	0.481
MDA (nmol/mL)	0.617	**<0.001**	**0.011**	0.505
Dityrosine (ng/mL)	0.495	**0.002**	0.407	0.099
Cytokines				
IFN-α2 (pg/mL)	0.379	0.136	0.142	0.891
IFN-γ (pg/mL)	**0.042**	0.594	0.721	0.909
IL-1β (pg/mL)	0.209	0.151	0.567	0.631
IL-6 (pg/mL)	0.607	**0.003**	**0.035**	0.326
IL-8 (pg/mL)	0.308	0.659	0.867	0.384
IL-10 (pg/mL)	0.220	**0.018**	**0.019**	0.418
IL-12p70 (pg/mL)	0.895	0.348	0.404	0.587
IL-17A (pg/mL)	0.816	0.079	0.760	0.714
IL-18 (pg/mL)	0.646	**<0.001**	0.261	0.995
IL-23 (pg/mL)	0.241	0.303	0.927	0.712
IL-33 (pg/mL)	0.343	0.369	0.200	0.700
MCP-1 (pg/mL)	0.575	**<0.001**	0.982	0.202
TNF-α (pg/mL)	0.214	0.791	0.822	0.403

*p*-values in bold are statistically significant

### Statistics

Data analyses were conducted using the open-source statistical software package R. Group differences between categorical data were determined through Fisher’s exact tests. Non-parametric Kruskal–Wallis H-tests and Mann–Whitney U-tests were employed to calculate group differences. Friedman’s test, along with post-hoc U-tests for repeated measures data, was applied to analyse the time course of oxidative stress, inflammatory markers and liver enzyme biomarkers within the CMZ and DIAZ treatment groups. To control for multiple hypothesis testing, Benjamini–Hochberg false discovery rate (FDR) p-value adjustment was utilized. A linear mixed-effects model (LMM) was employed to assess interactions between drug type and time, with liver enzymes, MDA, dityrosine, or inflammatory markers as dependent variables, drug type (CMZ or DIAZ) and time point (BL, D3, D5) as fixed factors, and blood alcohol concentration on admission serving as a covariate. CMZ was converted to DIAZ equivalents for statistical purposes (192 mg CMZ ≙ 5 mg DIAZ) [15]. All statistical tests were two-tailed with p < 0.05 considered significant.

## Results

### Baseline demographics, clinical and routine laboratory data

No significant differences were observed between the CMZ, DIAZ, and control groups in terms of age, sex, BMI, COPD, leukocyte count and CRP levels at baseline. The CMZ and DIAZ groups had significantly higher AUDIT and OCDS scores, and significantly more smokers compared to controls (p < 0.001, Table 1). Significant differences were observed among all biomarkers of alcohol consumption and liver damage, including BAC, EtG, Peth, CDT, GGT, ALAT, ASAT, ASAT/ALAT ratio (i.e. the de Ritis ratio) and MCV, indicating higher values in both CMZ and DIAZ groups compared to controls (p < 0.05, Table 1), without significant differences between CMD and DIAZ groups at baseline.

### Time course in CMZ and DIAZ treatment groups

#### (a) Duration of AWT, required drug dose and complications

Both treatments were comparable in terms of mean cumulative drug dose required [CMZ group: 42.5 (26.3;87.5) vs. DIAZ group: 45.0 (35.0;107.5) DIAZ equivalents (mg), p = 0.476] and complication rates [CMZ group: 1 of 25 vs. DIAZ group: 2 of 25 patients with delirium (haloperidol treatment), p = 1.000; CMZ group: 0 of 25 vs. DIAZ group: 2 of 25 patients with hypertensive crisis (clonidine or nitrendipine treatment), p = 0.490; CMZ group: 0 of 25 vs. DIAZ group: 1 of 25 with epileptic seizure, p = 1.000]. Mean AWT duration tended to be shorter in CMZ versus DIAZ group, but this did not reach statistical significance [74.3 (51.8;106.0) vs. 101.8 (74.0;133.7) h, p = 0.065]. No serious adverse events were observed for either treatment.

#### (b) Biomarkers of liver damage, oxidative stress and inflammation

Only the CMZ group showed significant changes over time in the biomarkers for liver damage GGT and ASAT, while the ASAT/ALAT ratio decreased over time in both the CMZ and DIAZ groups (Supplementary Table). However, no significant differences over the time were found between CMZ and DIAZ groups (Table 2**,** drug type X time interaction; Supplementary Figure).

MDA increased significantly over time on D3 and D5 compared to BL in the DIAZ group. However, MDA and dityrosine showed no significant differences among CMZ and DIAZ groups over time (drug type X time interaction).

IL-6, IL-10 and MCP-1 decreased significantly in DIAZ, and IL-18 was significantly decreased in both CMZ and DIAZ over time, with no significant differences between treatment groups at any time point.

## Discussion

Although not significant, our study showed that the CMZ group took approximately 24 h less to complete the AWT than the DIAZ group. This could be advantageous because patients without physical withdrawal symptoms are better able to participate in psychotherapeutic interventions. However, our main hypothesis that AWT with CMZ would lead to a quicker normalisation of oxidative stress, inflammatory cytokines and liver enzymes compared to DIAZ during the 5-day preservation period was not proven.

This finding may contrast in part to two studies by Hohmann et al. covering the same clinical trial, which demonstrated an advantage of CMZ over the benzodiazepine clorazepate regarding regression of CYP2E1 and ALAT [16, 17]. However, it should be noted that our naturalistic study covered the clinical course and laboratory parameters for only 3–5 days, while Hohmann et al. addressed a longer observation period of 7–10 days. Consequently, we cannot say whether a longer period of observation might have resulted in significant differences between medication groups. Furthermore, the previous findings with clorazepate might not be completely transferrable to DIAZ even though both drugs are classed as benzodiazepines.

The finding that the ASAT/ALAT ratio decreased over time under CMZ and DIAZ therapy may indicate a reduction in liver damage with each of these treatments [18]. Although some other markers of oxidative damage (MDA) and inflammation (IL-6, IL-10 and MCP-1) showed potential differences over the abbreviated time course, a daily and more extensive time course would be required to confirm these differences between the two treatment groups.

Considering these inconclusive results, we suggest the need for larger clinical studies comparing the effects of CMZ with those of multiple benzodiazepine compounds. In addition to assessment of potential adverse events, these studies should include measurements of CYP2E1 activity, as well as biomarkers of oxidation–reduction (REDOX), inflammation and liver damage. This will be important in the search for CYP2E1 inhibitors that are effective and safe to use in AWT over longer time periods.

More fundamentally, however, the question arises as to whether it would actually have a relevant influence on the medium to long-term clinical outcome if a drug is superior in terms of oxidative stress, inflammation and liver health during a few days of AWT, given the long-term accumulated liver damage caused by alcohol dependence.

## Supplementary Information

Below is the link to the electronic supplementary material.

Supplementary file1 (PPT 261 kb)

Supplementary file2 (DOC 206 kb)

## Data Availability

All data are either presented in the manuscript or supplementary materials. Additional information can be made available upon reasonable request.

## Abbreviations

ADH: Alcohol dehydrogenase
ALAT: Alanine-aminotransferase
ASAT: Aspartate-aminotransferase
AUDIT: Alcohol use disorders identification test
AWT: Alcohol withdrawal treatment
BAC: Blood alcohol concentration
BL: Baseline
BMI: Body mass index
CDT: Carbohydrate-deficient transferrin
CMZ: Clomethiazole treatment group
COPD: Chronic obstructive pulmonary disease
CRP: C-reactive protein
CYP2E1: Cytochrome P450 2E1
D3: Day 3
D5: Day 5
DIAZ: Diazepam treatment group
EtG: Ethyl glucuronide
FDR: False discovery rate (corrected p-value)
GGT: Gamma-glutamyltransferase
HPLC: High performance liquid chromatography
IFN: Interferon
IL: Interleukin
LOQ: Limit of quantification
LMM: Linear mixed-effects model
MCP-1: Monocyte chemotactic protein 1
MCV: Mean corpuscular volume
MDA: Malondialdehyde
OCDS: Obsessive compulsive drinking scale
PEth: Phosphatidylethanol
REDOX: Oxidation–reduction
ROS: Reactive oxygen species
TNF: Tumour necrosis factor

## Footnotes

1 According to the manufacturer Cheplapharm, clomethiazole is also approved for AWT in Cyprus, the Czech Republic, Hungary, Iceland, Norway, Poland, Slovenia, Spain, Sweden, Switzerland and the United Kingdom.

2 Out of 129 patients who consented to participate, 51 were excluded according to the above exclusion criteria after reviewing medical histories, results of physical examinations and blood tests. This gave a final study population of 78 patients, divided into CMZ (n = 26) and DIAZ (n = 37) treatment groups, and individuals with alcohol dependence who did not require medication during detoxification (n = 15). For this study, the two treatment groups were reduced further to 25 patients each to allow matching for sex, body mass index (BMI), age, smoking, chronic obstructive pulmonary disease (COPD) and age at first AWT.
