# Supplementary material for: Naturalistic comparison of clomethiazole and Diazepam treatment in alcohol withdrawal: effects on oxidative stress, inflammatory cytokines and hepatic biomarkers
Source: Eur Arch Psychiatry Clin Neurosci. 2024 Jun 8;275(2):573–9. doi: 10.1007/s00406-024-01835-7 (PMC11910401; doi:10.1007/s00406-024-01835-7)
Supplement: Supplementary file 1 — Supplementary file1 (PPT 261 kb) [file 406_2024_1835_MOESM1_ESM.doc]

**Supplementary Table:** Time course of liver enzymes, oxidative stress, cytokines and drug levels during AWT with CMZ and DIAZ. *Annotation:* Data are presented as median (quartile 1; quartile 3, number of cases). For the time courses, pairwise post hoc U-tests were only carried out if the entry test (Friedman) was positive for all time points. *Abbreviations:* BL, baseline; D3, day 3; D5, day 5; FDR, false discovery rate; LOQ, limit of quantification (15 ng/mL for CMZ; 1 ng/mL for DIAZ and Nordiazepam).

| **variables** | | **CMZ** | | **DIAZ** | | **test** | | **test value** | | **p-value** |
| --- | --- | --- | --- | --- | --- | --- | --- | --- | --- | --- |
| **Biomarkers of liver damage** | | | | | | | | | | |
| GGT (µmol/L) BL | | 2.44 (1.25;7.91;25) | | 1.96 (0.73;3.01;25) | | U-Test | | W=381.00 | | 0.187 |
| GGT (µmol/L) D3 | | 2.65 (1.35;8.66;25) | | 1.90 (0.94;3.29;24) | | U-Test | | W=362.00 | | 0.221 |
| GGT (µmol/L) D5 | | 3.32 (1.28;7.75;12) | | 1.72 (0.90;3.90;16) | | U-Test | | W=121.50 | | 0.246 |
| BL,D3,D5 test | | Friedman chi-squared=8.67 | | Friedman chi-squared=3.76 | |  | |  | |  |
| BL,D3,D5 p-value | | **p=0.013 (FDR)** | | p=0.152 (FDR) | |  | |  | |  |
| D3-BL p-value | | p=0.622 (FDR) | | - | |  | |  | |  |
| D5-BL p-value | | p=0.194 (FDR) | | - | |  | |  | |  |
| D5-D3 p-value | | **p=0.037 (FDR)** | | - | |  | |  | |  |
|  | |  | |  | |  | |  | |  |
| ALAT (µmol/L) BL | | 0.84 (0.55;1.31;25) | | 0.84 (0.53;0.96;25) | | U-Test | | W=353.00 | | 0.438 |
| ALAT (µmol/L) D3 | | 0.79 (0.48;1.27;25) | | 0.71 (0.56;1.04;25) | | U-Test | | W=322.50 | | 0.854 |
| ALAT (µmol/L) D5 | | 0.75 (0.51;1.56;12) | | 1.08 (0.57;1.35;16) | | U-Test | | W=83.00 | | 0.568 |
| BL,D3,D5 test | | Friedman chi-squared=3.36 | | Friedman chi-squared=0.67 | |  | |  | |  |
| BL,D3,D5 p-value | | p=0.186 (FDR) | | p=0.717 (FDR) | |  | |  | |  |
|  | |  | |  | |  | |  | |  |
| ASAT (µmol/L) BL | | 1.08 (0.77;1.64;25) | | 1.10 (0.63;2.17;25) | | U-Test | | W=318.50 | | 0.915 |
| ASAT (µmol/L) D3 | | 0.94 (0.63;1.58;25) | | 0.99 (0.59;1.87;25) | | U-Test | | W=315.00 | | 0.969 |
| ASAT (µmol/L) D5 | | 0.80 (0.66;2.24;12) | | 1.14 (0.53;2.31;16) | | U-Test | | W=84.00 | | 0.593 |
| BL,D3,D5 test | | Friedman chi-squared=9.23 | | Friedman chi-squared=4.03 | |  | |  | |  |
| BL,D3,D5 p-value | | **p=0.010 (FDR)** | | p=0.133 (FDR) | |  | |  | |  |
| D3-BL p-value | | p=0.138 (FDR) | | - | |  | |  | |  |
| D5-BL p-value | | **p=0.021 (FDR)** | | - | |  | |  | |  |
| D5-D3 p-value | | p=0.307 (FDR) | | - | |  | |  | |  |
|  | |  | |  | |  | |  | |  |
| ASAT/ALAT ratio BL | | 1.29 (1.05;1.92;25) | | 1.43 (1.15;2.01;25) | | U-Test | | W=279.50 | | 0.528 |
| ASAT/ALAT ratio D3 | | 1.25 (1.04;1.80;25) | | 1.28 (1.10;1.53;25) | | U-Test | | W=312.50 | | 1.000 |
| ASAT/ALAT ratio D5 | | 1.16 (0.97;1.55;12) | | 1.19 (1.00;1.53;16) | | U-Test | | W=97.00 | | 0.982 |
| BL,D3,D5 test | | Friedman chi-squared=8.67 | | Friedman chi-squared=13.88 | |  | |  | |  |
| BL,D3,D5 p-value | | **p=0.013 (FDR)** | | **p<0.001 (FDR)** | |  | |  | |  |
| D3-BL p-value | | p=0.077 (FDR) | | **p=0.006 (FDR)** | |  | |  | |  |
| D5-BL p-value | | **p=0.010 (FDR)** | | **p=0.001 (FDR)** | |  | |  | |  |
| D5-D3 p-value | | **p=0.031 (FDR)** | | **p=0.008 (FDR)** | |  | |  | |  |
|  | |  | |  | |  | |  | |  |
| **Biomarkers of oxidative stress** | | | | | | | | | | |
| MDA (nmol/mL) BL | | 1.11 (1.01;1.41;18) | | 1.06 (0.88;1.33;16) | | U-Test | | W=170.00 | | 0.379 |
| MDA (nmol/mL) D3 | | 1.58 (1.32;1.77;19) | | 1.52 (1.40;1.67;16) | | U-Test | | W=165.50 | | 0.667 |
| MDA (nmol/mL) D5 | | 1.62 (1.31;1.83;9) | | 1.46 (1.38;1.67;10) | | U-Test | | W=47.50 | | 0.870 |
| BL,D3,D5 test | | Friedman chi-squared=4.29 | | Friedman chi-squared=12.60 | |  | |  | |  |
| BL,D3,D5 p-value | | p=0.117 (FDR) | | **p=0.002 (FDR)** | |  | |  | |  |
| D3-BL p-value | | - | | **p=0.016 (FDR)** | |  | |  | |  |
| D5-BL p-value | | - | | **p=0.006 (FDR)** | |  | |  | |  |
| D5-D3 p-value | | - | | p=0.241 (FDR) | |  | |  | |  |
|  | |  | |  | |  | |  | |  |
| Dityrosine (ng/mL) BL | | 27.26 (12.94;44.30;19) | | 41.20 (19.82;80.09;16) | | U-Test | | W=104.00 | | 0.117 |
| Dityrosine (ng/mL) D3 | | 62.70 (36.63;79.13;18) | | 81.50 (50.39;115.75;15) | | U-Test | | W=100.00 | | 0.215 |
| Dityrosine (ng/mL) D5 | | 53.56 (33.77;84.75;9) | | 43.54 (22.95;70.89;10) | | U-Test | | W=54.00 | | 0.497 |
| BL,D3,D5 test | | Friedman chi-squared=3.56 | | Friedman chi-squared=3.80 | |  | |  | |  |
| BL,D3,D5 p-value | | p=0.169 (FDR) | | p=0.150 (FDR) | |  | |  | |  |
|  | |  | |  | |  | |  | |  |
| **Cytokines** | | | | | | | | | | |
| IFN-α2 (pg/mL) BL | | 8.29 (5.64;11.20;25) | | 8.53 (6.10;14.22;25) | | U-Test | | W=273.00 | | 0.453 |
| IFN-α2 (pg/mL) D3 | | 6.75 (5.49;14.05;25) | | 9.23 (6.09;15.24;25) | | U-Test | | W=275.00 | | 0.473 |
| IFN-α2 (pg/mL) D5 | | 7.97 (2.56;10.68;12) | | 7.56 (5.69;16.29;16) | | U-Test | | W=80.00 | | 0.478 |
| BL,D3,D5 test | | Friedman chi-squared=2.17 | | Friedman chi-squared=1.63 | |  | |  | |  |
| BL,D3,D5 p-value | | p=0.338 (FDR) | | p=0.444 (FDR) | |  | |  | |  |
|  | |  | |  | |  | |  | |  |
| IFN-γ (pg/mL) BL | | 7.24 (4.56;13.17;25) | | 10.91 (7.19;24.61;25) | | U-Test | | W=230.00 | | 0.112 |
| IFN-γ (pg/mL) D3 | | 8.05 (4.87;15.91;25) | | 11.55 (6.51;17.52;24) | | U-Test | | W=241.00 | | 0.242 |
| IFN-γ (pg/mL) D5 | | 6.42 (4.33;12.14;12) | | 11.97 (7.58;16.29;16) | | U-Test | | W=60.00 | | 0.099 |
| BL,D3,D5 test | | Friedman chi-squared=1.26 | | Friedman chi-squared=1.50 | |  | |  | |  |
| BL,D3,D5 p-value | | p=0.534 (FDR) | | p=0.472 (FDR) | |  | |  | |  |
|  | |  | |  | |  | |  | |  |
| IL-1ß (pg/mL) BL | | 20.09 (10.49;48.84;25) | | 25.50 (13.88;80.18;25) | | U-Test | | W=246.00 | | 0.200 |
| IL-1ß (pg/mL) D3 | | 19.80 (12.98;64.82;25) | | 26.44 (17.28;71.36;25) | | U-Test | | W=263.50 | | 0.347 |
| IL-1ß (pg/mL) D5 | | 20.26 (7.60;69.04;12) | | 25.55 (9.84;60.98;16) | | U-Test | | W=86.50 | | 0.676 |
| BL,D3,D5 test | | Friedman chi-squared=0.73 | | Friedman chi-squared=0.57 | |  | |  | |  |
| BL,D3,D5 p-value | | p=0.695 (FDR) | | p=0.751 (FDR) | |  | |  | |  |
|  | |  | |  | |  | |  | |  |
| IL-6 (pg/mL) BL | | 19.23 (13.00;28.52;25) | | 22.52 (10.84;29.78;25) | | U-Test | | W=300.00 | | 0.818 |
| IL-6 (pg/mL) D3 | | 20.20 (14.17;35.17;25) | | 26.01 (12.62;44.85;25) | | U-Test | | W=277.00 | | 0.500 |
| IL-6 (pg/mL) D5 | | 16.26 (13.61;26.00;12) | | 18.36 (10.54;33.92;16) | | U-Test | | W=96.00 | | 1.000 |
| BL,D3,D5 test | | Friedman chi-squared=2.17 | | Friedman chi-squared=7.63 | |  | |  | |  |
| BL,D3,D5 p-value | | p=0.338 (FDR) | | **p=0.022 (FDR)** | |  | |  | |  |
| D3-BL p-value | | - | | p=0.140 (FDR) | |  | |  | |  |
| D5-BL p-value | | - | | p=0.860 (FDR) | |  | |  | |  |
| D5-D3 p-value | | - | | **p=0.004 (FDR)** | |  | |  | |  |
|  | |  | |  | |  | |  | |  |
| IL-8 (pg/mL) BL | | 59.94 (22.50;91.14;25) | | 60.93 (21.14;111.96;25) | | U-Test | | W=302.50 | | 0.854 |
| IL-8 (pg/mL) D3 | | 56.60 (22.55;85.71;25) | | 57.90 (27.82;102.45;25) | | U-Test | | W=284.00 | | 0.587 |
| IL-8 (pg/mL) D5 | | 28.93 (18.52;66.02;12) | | 66.75 (38.08;117.06;16) | | U-Test | | W=54.00 | | 0.053 |
| BL,D3,D5 test | | Friedman chi-squared=1.27 | | Friedman chi-squared=0.50 | |  | |  | |  |
| BL,D3,D5 p-value | | p=0.529 (FDR) | | p=0.779 (FDR) | |  | |  | |  |
|  | |  | |  | |  | |  | |  |
| IL-10 (pg/mL) BL | | 17.36 (14.15;24.40;25) | | 17.88 (12.12;27.48;25) | | U-Test | | W=280.00 | | 0.538 |
| IL-10 (pg/mL) D3 | | 19.84 (11.95;25.98;25) | | 23.47 (13.20;37.63;25) | | U-Test | | W=260.00 | | 0.316 |
| IL-10 (pg/mL) D5 | | 16.77 (12.50;21.24;12) | | 18.41 (8.45;27.09;16) | | U-Test | | W=88.00 | | 0.732 |
| BL,D3,D5 test | | Friedman chi-squared=4.67 | | Friedman chi-squared=8.22 | |  | |  | |  |
| BL,D3,D5 p-value | | p=0.097 (FDR) | | **p=0.016 (FDR)** | |  | |  | |  |
| D3-BL p-value | | - | | p=0.074 (FDR) | |  | |  | |  |
| D5-BL p-value | | - | | p=0.629 (FDR) | |  | |  | |  |
| D5-D3 p-value | | - | | **p=0.039 (FDR)** | |  | |  | |  |
|  | |  | |  | |  | |  | |  |
| IL-12p70 (pg/mL) BL | | 7.395 (5.395;9.592;25) | | 9.005 (4.930;11.523;25) | | U-Test | | W=281.00 | | 0.551 |
| IL-12p70 (pg/mL) D3 | | 7.67 (4.20;12.80;25) | | 8.48 (4.77;11.65;25) | | U-Test | | W=319.00 | | 0.908 |
| IL-12p70 (pg/mL) D5 | | 9.13 (4.62;11.52;12) | | 6.30 (3.05;10.77;16) | | U-Test | | W=119.00 | | 0.302 |
| BL,D3,D5 test | | Friedman chi-squared=2.17 | | Friedman chi-squared=2.87 | |  | |  | |  |
| BL,D3,D5 p-value | | p=0.338 (FDR) | | p=0.238 (FDR) | |  | |  | |  |
|  | |  | |  | |  | |  | |  |
| IL-17A (pg/mL) BL | | 2.52 (1.19;3.63;25) | | 1.84 (1.39;3.43;25) | | U-Test | | W=315.00 | | 0.969 |
| IL-17A (pg/mL) D3 | | 2.48 (0.80;3.92;25) | | 2.32 (1.42;3.99;25) | | U-Test | | W=298.50 | | 0.793 |
| IL-17A (pg/mL) D5 | | 1.75 (0.46;2.44;12) | | 1.91 (1.11;3.10;16) | | U-Test | | W=77.00 | | 0.390 |
| BL,D3,D5 test | | Friedman chi-squared=3.56 | | Friedman chi-squared=1.81 | |  | |  | |  |
| BL,D3,D5 p-value | | p=0.169 (FDR) | | p=0.405 (FDR) | |  | |  | |  |
|  | |  | |  | |  | |  | |  |
| IL-18 (pg/mL) BL | | 813.2 (561.6;1105.6;25) | | 702.8 (450.6;1057.3;25) | | U-Test | | W=357.00 | | 0.397 |
| IL-18 (pg/mL) D3 | | 676.4 (518.7;994.8;25) | | 560.3 (402.0;957.6;25) | | U-Test | | W=363.00 | | 0.335 |
| IL-18 (pg/mL) D5 | | 783.6 (491.9;1060.8;12) | | 660.6 (354.7;1023.3;16) | | U-Test | | W=115.00 | | 0.397 |
| BL,D3,D5 test | | Friedman chi-squared=7.17 | | Friedman chi-squared=12.88 | |  | |  | |  |
| BL,D3,D5 p-value | | **p=0.028 (FDR)** | | **p=0.002 (FDR)** | |  | |  | |  |
| D3-BL p-value | | p=0.301 (FDR) | | **p=0.032 (FDR)** | |  | |  | |  |
| D5-BL p-value | | **p=0.015 (FDR)** | | **p<0.001 (FDR)** | |  | |  | |  |
| D5-D3 p-value | | p=0.301 (FDR) | | p=0.348 (FDR) | |  | |  | |  |
|  | |  | |  | |  | |  | |  |
| IL-23 (pg/mL) BL | | 16.57 (5.69;30.63;25) | | 25.36 (18.48;36.01;25) | | U-Test | | W=212.00 | | 0.052 |
| IL-23 (pg/mL) D3 | | 16.88 (6.57;31.08;25) | | 25.02 (16.23;42.44;25) | | U-Test | | W=244.00 | | 0.187 |
| IL-23 (pg/mL) D5 | | 16.54 (4.53;32.55;12) | | 24.13 (16.49;39.23;16) | | U-Test | | W=76.00 | | 0.365 |
| BL,D3,D5 test | | Friedman chi-squared=0.67 | | Friedman chi-squared=0.22 | |  | |  | |  |
| BL,D3,D5 p-value | | p=0.717 (FDR) | | p=0.895 (FDR) | |  | |  | |  |
|  | |  | |  | |  | |  | |  |
| IL-33 (pg/mL) BL | | 151.6 (59.7;262.9;25) | | 192.1 (82.2;381.0;25) | | U-Test | | W=247.00 | | 0.207 |
| IL-33 (pg/mL) D3 | | 127.0 (62.5;307.6;25) | | 205.2 (94.9;410.0;25) | | U-Test | | W=270.00 | | 0.415 |
| IL-33 (pg/mL) D5 | | 155.7 (36.2;319.4;12) | | 141.1 (93.1;445.6;16) | | U-Test | | W=72.00 | | 0.280 |
| BL,D3,D5 test | | Friedman chi-squared=2.28 | | Friedman chi-squared=0.50 | |  | |  | |  |
| BL,D3,D5 p-value | | p=0.320 (FDR) | | p=0.779 (FDR) | |  | |  | |  |
|  | |  | |  | |  | |  | |  |
| MCP-1 (pg/mL) BL | | 391.1 (191.6;725.0;25) | | 425.6 (264.2;624.2;25) | | U-Test | | W=318.00 | | 0.923 |
| MCP-1 (pg/mL) D3 | | 438.2 (281.7;827.3;25) | | 617.6 (371.6;819.2;25) | | U-Test | | W=279.00 | | 0.525 |
| MCP-1 (pg/mL) D5 | | 433.7 (202.0;691.5;12) | | 552.1 (170.7;729.9;16) | | U-Test | | W=89.00 | | 0.767 |
| BL,D3,D5 test | | Friedman chi-squared=2.67 | | Friedman chi-squared=7.13 | |  | |  | |  |
| BL,D3,D5 p-value | | p=0.264 (FDR) | | **p=0.028 (FDR)** | |  | |  | |  |
| D3-BL p-value | | - | | **p=0.027 (FDR)** | |  | |  | |  |
| D5-BL p-value | | - | | p=0.093 (FDR) | |  | |  | |  |
| D5-D3 p-value | | - | | **p=0.027 (FDR)** | |  | |  | |  |
|  | |  | |  | |  | |  | |  |
| TNF-α (pg/mL) BL | | 9.25 (4.72;19.62;25) | | 10.08 (8.60;18.03;22) | | U-Test | | W=248.00 | | 0.571 |
| TNF-α (pg/mL) D3 | | 12.00 (6.75;18.87;25) | | 11.44 (8.51;26.68;23) | | U-Test | | W=248.00 | | 0.420 |
| TNF-α (pg/mL) D5 | | 8.95 (4.27;21.40;12) | | 10.38 (9.25;33.85;15) | | U-Test | | W=66.00 | | 0.250 |
| BL,D3,D5 test | | Friedman chi-squared=1.20 | | Friedman chi-squared=0.60 | |  | |  | |  |
| BL,D3,D5 p-value | | p=0.549 (FDR) | | p=0.741 (FDR) | |  | |  | |  |
|  | |  | |  | |  | |  | |  |
| **Drug levels** | | | | | | | | | | |
| CMZ (ng/mL) BL | <LOQ (<LOQ;<LOQ;24) | | - | | - | | - | |  | |
| CMZ (ng/mL) D3 | 66 (<LOQ;362;25) | | - | | - | | - | |  | |
| CMZ (ng/mL) D5 | <LOQ (<LOQ;33;12) | | - | | - | | - | |  | |
| BL,D3,D5 test | Friedman chi-squared=18.59 | |  | |  | |  | |  | |
| BL,D3,D5 p-value | **p<0.001 (FDR)** | |  | |  | |  | |  | |
| D3-BL p-value | **p=0.009 (FDR)** | |  | |  | |  | |  | |
| D5-BL p-value | p=0.100 (FDR) | |  | |  | |  | |  | |
| D5-D3 p-value | **p=0.009 (FDR)** | |  | |  | |  | |  | |
|  |  | |  | |  | |  | |  | |
| DIAZ (ng/mL) BL | <LOQ (<LOQ;<LOQ;22) | | <LOQ (<LOQ;24;25) | | - | |  | |  | |
| DIAZ (ng/mL) D3 | <LOQ (<LOQ;2;9) | | 187 (114;415;25) | | - | |  | |  | |
| DIAZ (ng/mL) D5 | <LOQ (<LOQ;4;4) | | 143 (38;266;16) | | - | |  | |  | |
| BL,D3,D5 test |  | | Friedman chi-squared=19.63 | |  | |  | |  | |
| BL,D3,D5 p-value |  | | **p<0.001 (FDR)** | |  | |  | |  | |
| D3-BL p-value |  | | **p<0.001 (FDR)** | |  | |  | |  | |
| D5-BL p-value |  | | **p=0.003 (FDR)** | |  | |  | |  | |
| D5-D3 p-value |  | | **p=0.003 (FDR)** | |  | |  | |  | |
|  |  | |  | |  | |  | |  | |
| Nordiazepam (ng/mL) BL | <LOQ (<LOQ; <LOQ;22) | | <LOQ (<LOQ;20;25) | |  | |  | |  | |
| Nordiazepam (ng/mL) D3 | <LOQ (<LOQ;8;9) | | 100 (38;167;25) | |  | |  | |  | |
| Nordiazepam (ng/mL) D5 | <LOQ (<LOQ;33;4) | | 134 (79;269;16) | |  | |  | |  | |
| BL,D3,D5 test |  | | Friedman chi-squared=27.13 | |  | |  | |  | |
| BL,D3,D5 p-value |  | | **p<0.001 (FDR)** | |  | |  | |  | |
| D3-BL p-value |  | | **p<0.001 (FDR)** | |  | |  | |  | |
| D5-BL p-value |  | | **p<0.001 (FDR)** | |  | |  | |  | |
| D5-D3 p-value |  | | **p=0.006 (FDR)** | |  | |  | |  | |
