# Supplementary material for: Naturalistic comparison of clomethiazole and Diazepam treatment in alcohol withdrawal: effects on oxidative stress, inflammatory cytokines and hepatic biomarkers
Source: Eur Arch Psychiatry Clin Neurosci. 2024 Jun 8;275(2):573–9. doi: 10.1007/s00406-024-01835-7 (PMC11910401; doi:10.1007/s00406-024-01835-7)
Supplement: Supplementary file 2 — Supplementary file2 (DOC 206 kb) [file 406_2024_1835_MOESM2_ESM.ppt]

## Slide 1
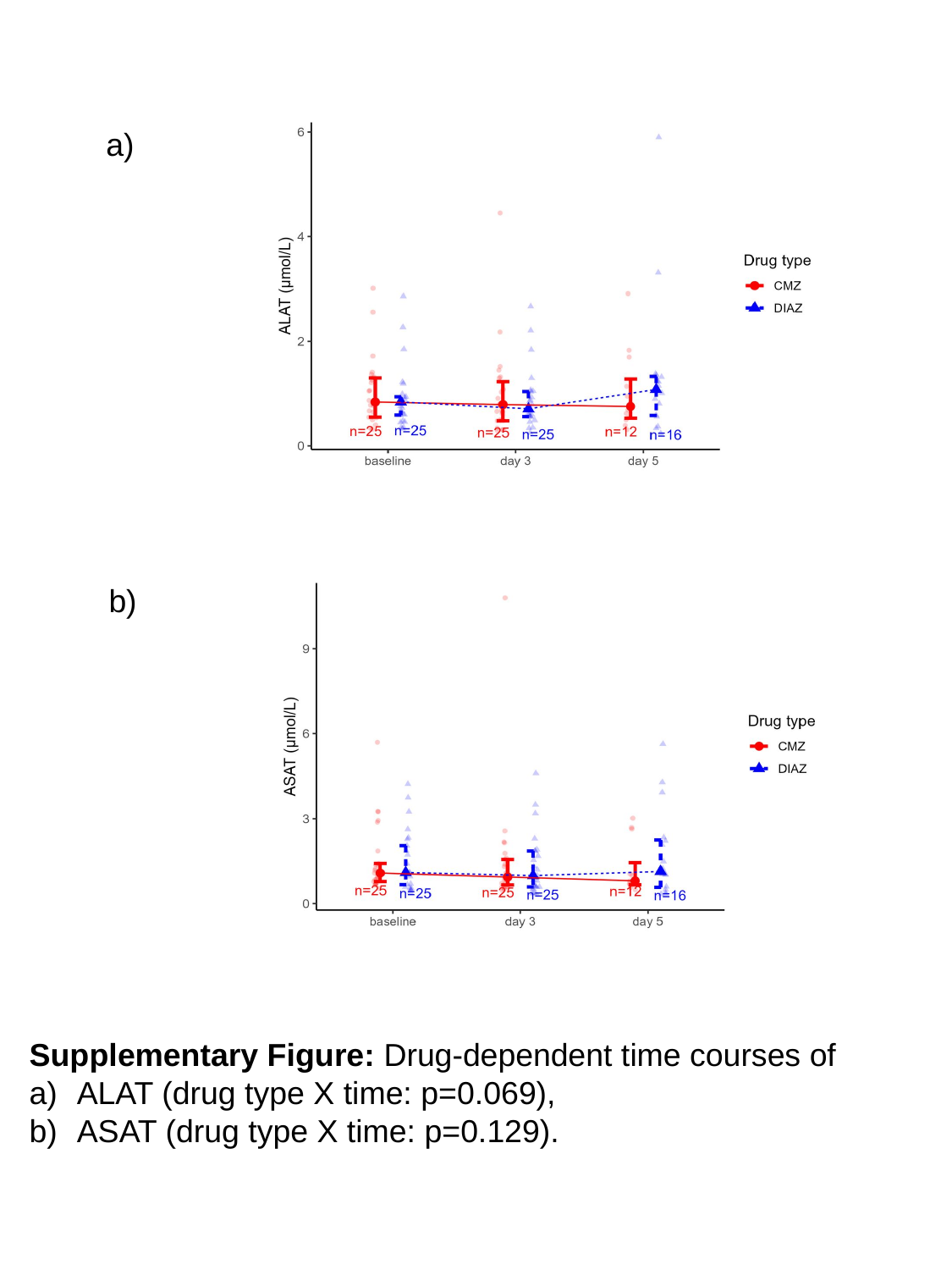

a)
b)
Supplementary Figure: Drug-dependent time courses of
ALAT (drug type X time: p=0.069),
ASAT (drug type X time: p=0.129).
